# Supplementary material for: Mechanism of NanR gene repression and allosteric induction of bacterial sialic acid metabolism
Source: Nat Commun. 2021 Mar 31;12:1988. doi: 10.1038/s41467-021-22253-6 (PMC8012715; doi:10.1038/s41467-021-22253-6)
Supplement: Supplementary file 1 — Supplementary Information [file 41467_2021_22253_MOESM1_ESM.pdf]

SUPPLEMENTARY INFORMATION

Mechanism of NanR gene repression and allosteric induction of bacterial sialic acid metabolism

Horne, Venugopal et al

SUPPLEMENTARY FIGURES AND LEGENDS

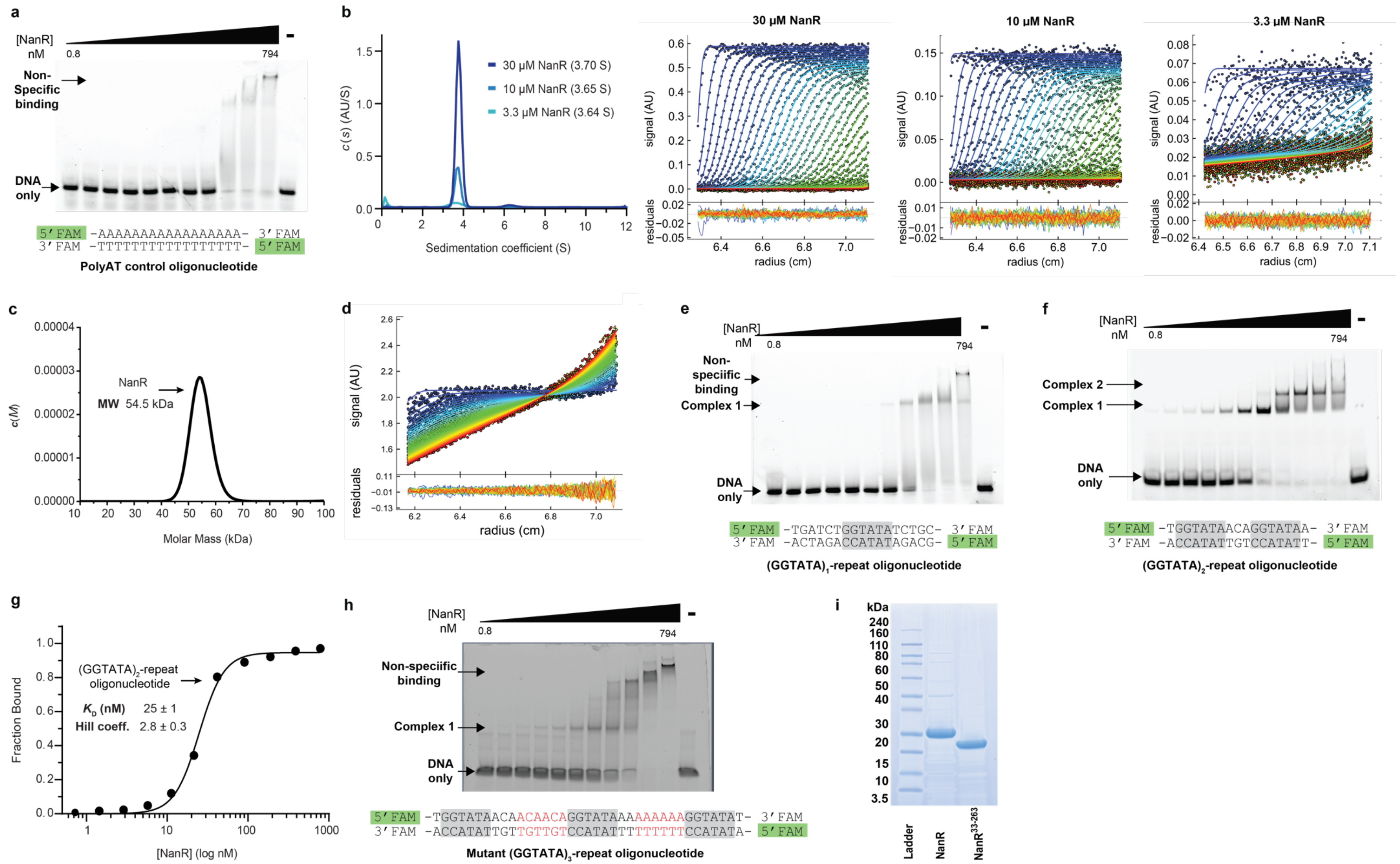

Supplementary Figure 1. NanR-DNA interaction. a, Specific DNA binding is abolished in the poly adenine/thymine (poly-AT) control oligonucleotide. At high concentrations (>200 nM), non-specific binding is observed. **b,** Sedimentation velocity data for NanR at 3.3-30 µM is a single monodisperse species at 3.64-3.70 S. The raw data and residual fits are shown to the right of the pane, where every third scan is shown. The data are fit to a continuous size [*c*(*s*)] distribution, as implemented in SEDFIT[1]. The hydrodynamic properties for the fit are listed in **Supplementary Table 1. c,** Sedimentation velocity data for NanR at 10 µM fit to a continuous mass [*c*(M)] distribution giving a molar mass of 54.5-kDa across the peak, consistent with the calculated mass of the NanR dimer (59.0-kDa). The RMSD for the fit was 0.004. **d,** When FAM alone is mixed with NanR at 10 µM and sedimentation velocity data is collected at 50,000 rpm and 495 nm (monitoring only FAM), the FAM molecule is not transported with the protein. This control experiment demonstrates that NanR does not bind the FAM label. **e,** Poor DNA binding activity is observed with a single GGTATA repeat, suggesting the regulation mechanism is dependent on cooperative binding. **f,** In contrast, two concentration-dependent complexes are observed when two GGTATA repeats are present. The concentration of DNA in the experiments **e** and **f** is 10 nM. **g,** The binding isotherm from the $(GGTATA)_2$-repeat oligonucleotide with NanR. The data was best fit to a Hill equation (AIC value of 99%), when compared with the non-cooperative binding model (AIC value of 1%). The mean + SD of the K_D and Hill coefficient is shown. **h,** Increasing the length of the spacer between the GGTATA repeats altered NanR binding. The additional nucleotides are highlighted red in the mutant $(GGTATA)_3$-repeat oligonucleotide. The concentration of DNA in the experiment is 10 nM. **i,** SDS-PAGE analysis showing protein molecular weight ladder (Lane 1), and purified *E. coli* NanR (Lane 2) and $NanR^{33–263}$ (Lane 3) following size exclusion chromatography. The purity of the shown protein preparations are representative of at least 3 independent expressions and purifications for each construct. Molecular weight markers were not required in the EMSA gels (a, e-f, h), since these experiments were used to determine the response of titrated protein with respect to a fixed concentration of DNA, which allowed us to estimate the dissociation constant. The size, or better the mass, of the various protein-DNA complexes was determined using analytical ultracentrifugation, as shown in **Fig. 3** and **Supplementary Table 5.** The EMSA gels shown are representative of at least 3 independent experiments for each oligonucleotide.

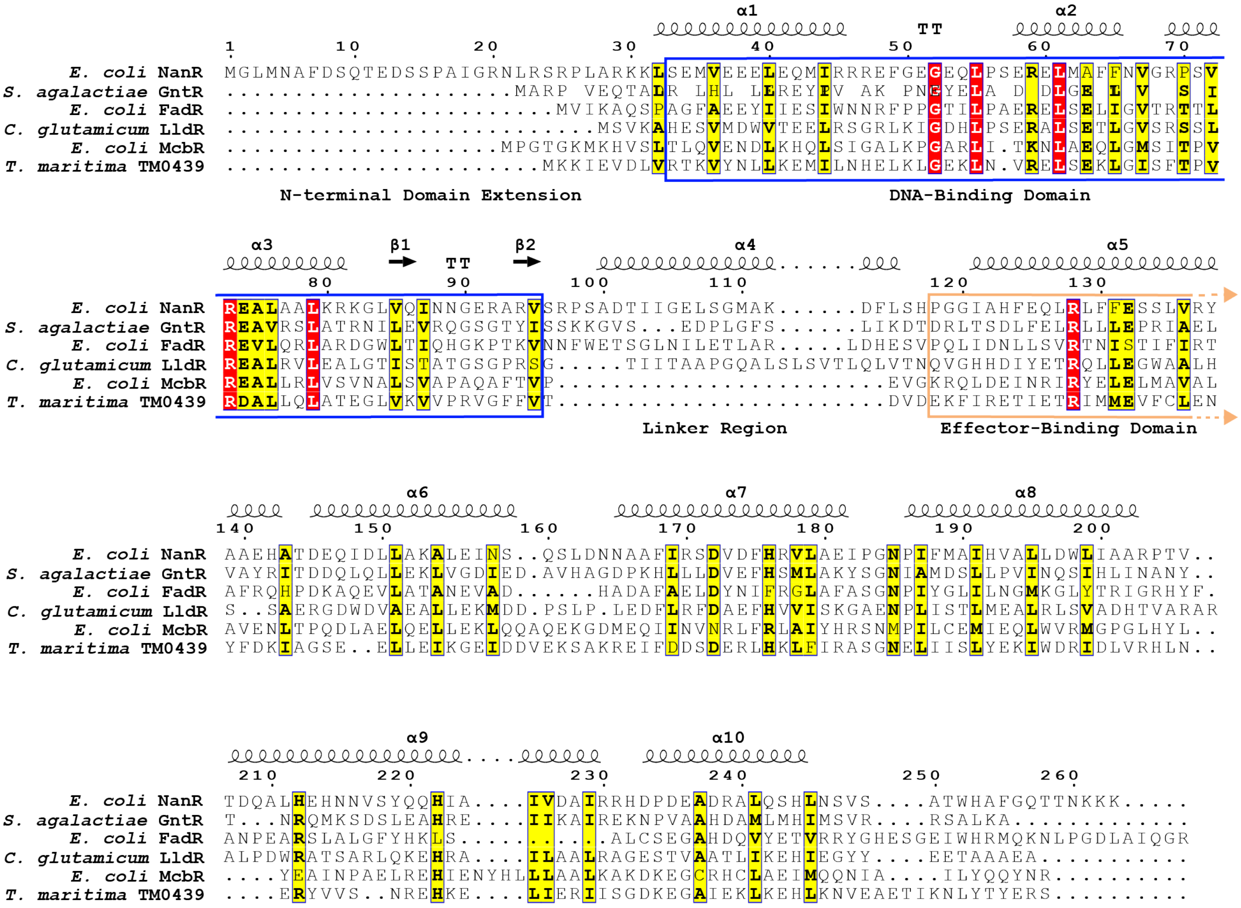

Supplementary Figure 2. NanR has a longer N-terminal sequence, when compared to known GntR proteins. Multiple sequence alignment performed using Clustal Omega[2] and generated using ESPript 3.0[3]. The top five hits from a sequence homology search within the PDB were used in the sequence alignment: *S. agalactiae* GntR – PDB ID 6AZ6[4]; *E. coli* FadR – PDB ID 1E2X[5]; *C. glutamicum* LldR – PDB ID 2DI3[6]; *E. coli* McbR – PDB ID 4P9F[7]; *T. maritima* TM0439 – PDB ID 3FMS[8]. The red background highlights identical residues, while the yellow background indicates similar residues. Secondary structure elements depicted above the alignment were generated from the crystal structure of *E. coli* NanR (**Fig. 4**), solved in this study. The N-terminal DNA-binding domain (blue box), the linker region, and the C-terminal effector-binding domain (beige box) are highlighted.

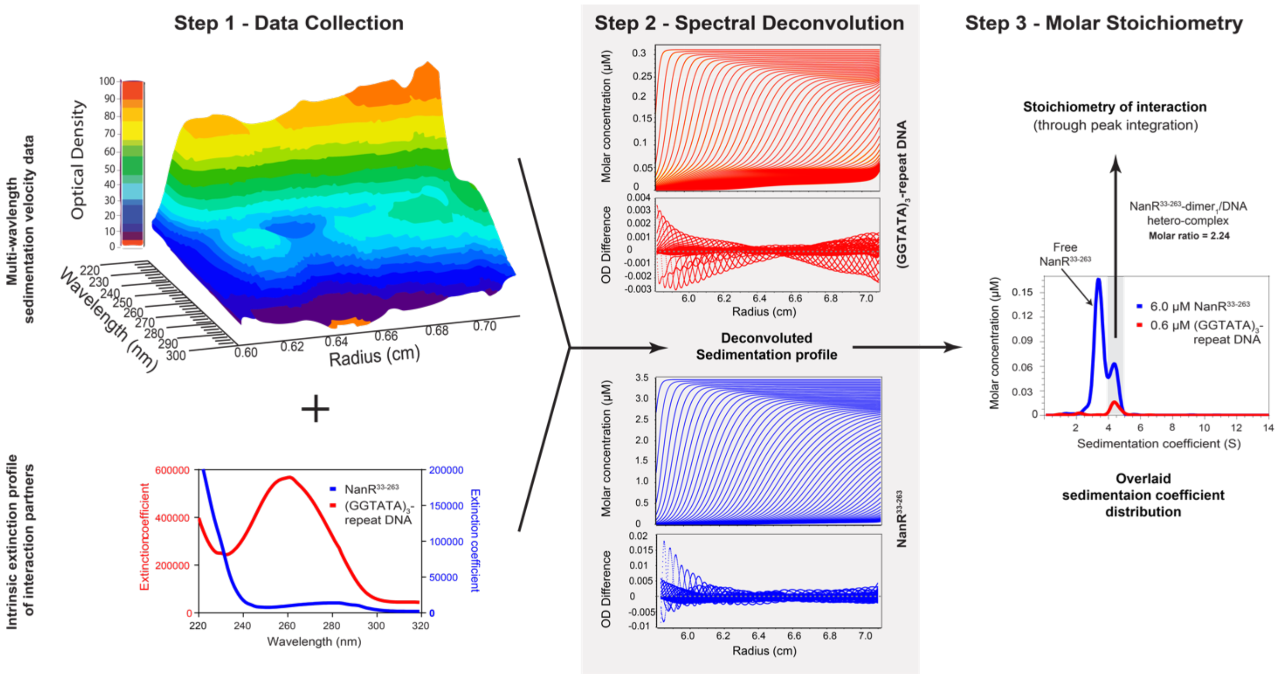

Supplementary Figure 3. Multiwavelength sedimentation velocity data for NanR$^{33–263}$ and DNA and analysis workflow. ***Step 1,*** four-dimensional multiwavelength sedimentation velocity data (top left) is collected across a range of wavelengths (220–300 nm). Only one time point is presented, highlighting peaks at 220 nm and 260–280 nm, consistent with a mixture of protein and DNA. An intrinsic extinction profile (bottom left) is generated for each interaction partner by globally fitting a dilution series of absorbance profiles using UltraScan[9]. ***Step 2,*** the intrinsic extinction profile is then used to spectrally deconvolute the multiwavelength data into separate sedimentation profiles for each interacting component (NanR$^{33–263}$ in red, (GGTATA)$_3$-repeat DNA in blue) by employing the non-negatively constrained least squares algorithm[9,10]. This process also scales the data to molar concentrations. ***Step 3,*** once deconvoluted and on a molar scale, the stoichiometry of the complex can simply be extracted by integrating the molar ratio of the co-migrating peaks (shaded box). In addition to this spectral characterization, the hydrodynamic information can also be accessed, providing such parameters as the molar mass and frictional ratio of each species. Together this enables interacting species to be characterized with high resolution.

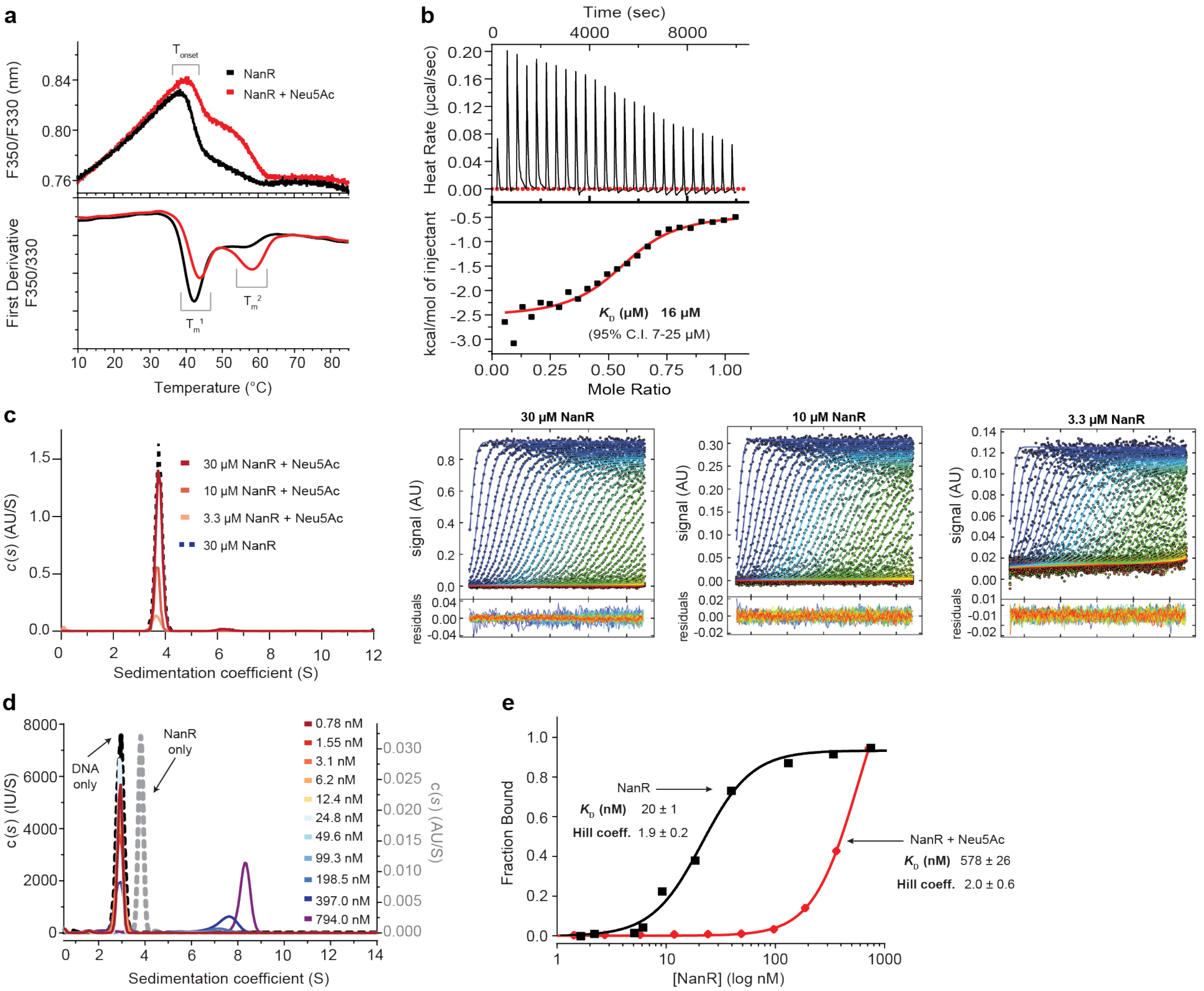

Supplementary Figure 4. Binding interaction between NanR and Neu5Ac and effect on DNA binding. a, Thermal stability of NanR (33 μM in black) with and without Neu5Ac (20 mM in red) using differential scanning fluorimetry. The results are presented as the ratio of intrinsic tryptophan fluorescence at the emission wavelengths of 350 and 330 nm (top pane) and the first derivative of this fluorescence ratio (lower pane). An increase in the thermal stability of NanR is observed in the presence of Neu5Ac at both the T_{onset} and the first transition melting temperature (T_m^1), which verifies Neu5Ac can bind NanR. The second transition melting temperature (T_m^2), observed only in the presence of Neu5Ac, may reflect increased thermal stability of the C-terminal effector-binding domain of NanR when it binds Neu5Ac. **b,** ITC isotherm of Neu5Ac (1 mM) titrated into NanR (416 μM) in buffer C at 8 °C. The titration involved 25 injections of ligand solution (2 μL) into the protein sample cell with a 200 sec interval between subsequent injections. When fitted, the data gave a K_D value of 16 μM. Data represent mean and 95% confidence interval of two independent experiments. **c,** Sedimentation velocity data for NanR in the presence of Neu5Ac (20 mM) at 3.3–30 μM is a single monodisperse species at 3.64–3.70 S. The raw data and residual fits are shown to the right of the pane, where every third scan is shown. The data is fit to a continuous size [*c*(*s*)] distribution, as implemented in SEDFIT[1]. The hydrodynamic properties for the fit are listed in **Supplementary Table 1. d,** Continuous sedimentation coefficient [c(s)] distributions of NanR (0.8–794 nM) titrated against the FAM-5′-labeled $(GGTATA)_3$-repeat (80 nM) in the presence of Neu5Ac (20 mM). The fitted parameters are shown in **Supplementary Table 6.** Sedimentation data performed using the same setup in the absence of Neu5Ac is shown in Fig. 2c. **e,** Binding isotherm data from the fluorescence-detection analytical ultracentrifugation (AUC) experiments for NanR titrated against the FAM-5′-labeled $(GGTATA)_3$-repeat in presence (red line) and absence (black line) of Neu5Ac. Data best fitted the Hill model (AIC value of 99%), as opposed to a non-cooperative binding model (AIC value of 1%). The mean ± SD of the K_D and Hill coefficient are shown in both.

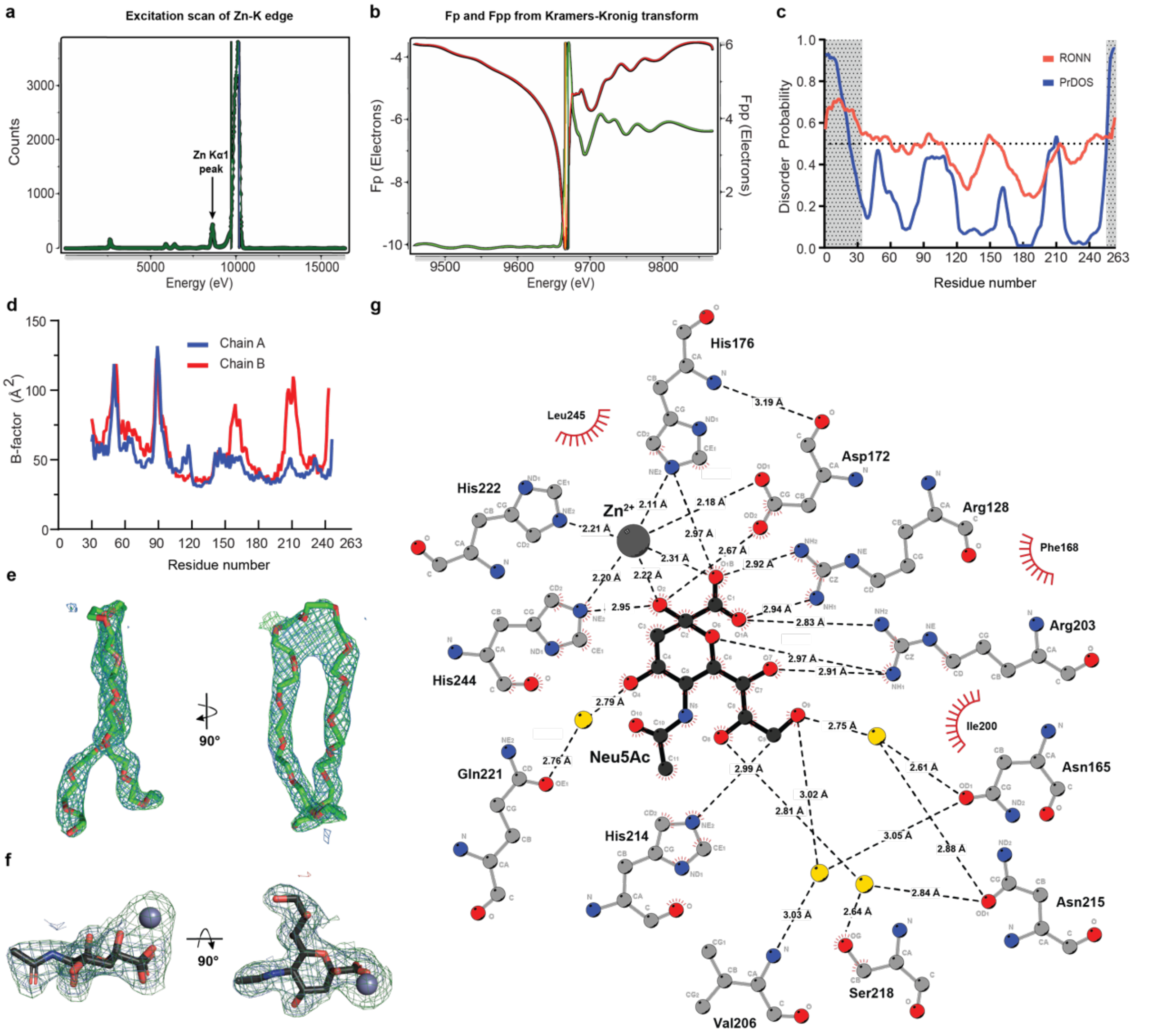

Supplementary Figure 5. Detection of elemental zinc, disorder prediction, and ligand analysis of the crystal structure. a, Elemental analysis of the NanR crystal using X-ray fluorescence suggests the presence of zinc (Zn Kα_1 peak corresponding to an emission energy of ~8.6 KeV[11]). **b,** MAD scan of the NanR crystal. Derivation of f' (red) and f'' (green) from the normalized MAD scan was calculated using the Kramers-Kronig transform in Chooch[12] The absorption edge of Zn corresponds to 9670.10 eV and the inflection energy to 9665.92 eV. **c,** Disorder probability for NanR estimated from the RONN (https://www.strubi.ox.ac.uk/RONN) and PrDOS (http://prdos.hgc.jp/cgi-bin/top.cgi) online servers. The regions of disorder are highlighted (grey with dot). **d.** Plot of B-factor values for chains A (Neu5Ac/Zn^{2+} bound, blue) and B (ligand free, red) of the NanR crystal structure. **e,** Omit map showing the electron density for polyethylene glycol. Here, the 2F*o*-F*c* electron density map (1.0 σ, blue mesh) and the mF*o*-F*c* omit electron density map (3.0 σ, green mesh) is shown. **f,** Omit map showing the electron density for Neu5Ac in its β-anomeric form (depicted as sticks) and Zn^{2+} (grey sphere). Here, the 2F*o*-F*c* electron density map (1.0 σ, blue mesh) and the mF*o*-F*c* omit electron density map (3.0 σ, green mesh) is shown. **g,** Schematic representation of the NanR-Neu5Ac-Zn^{2+} interaction network. The hydrogen-bonded network is presented (black dashed lines) and the distances between partners are labeled. Key interacting water molecules (yellow spheres) and hydrophobic contacts (red arcs with spokes) are shown. Figure generated by LigPlot+[13].

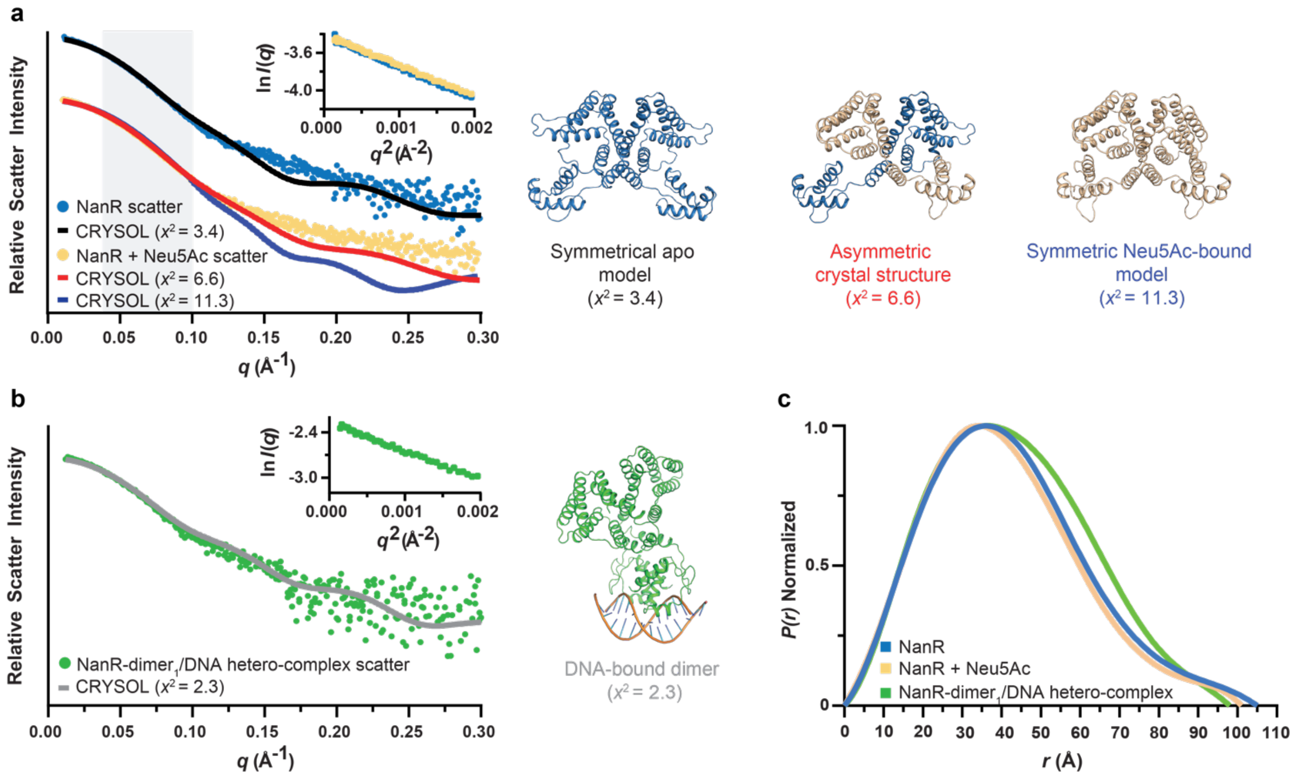

Supplementary Figure 6. Small angle X-ray scattering experiments of *E. coli* NanR and the NanR-DNA hetero-complex. a, Small angle X-ray scattering data for NanR alone (blue) and NanR in the presence of Neu5Ac (beige). The difference at low q is highlighted in the shaded area. The scattering data for NanR (blue) is best fit (black line, X^2=3.4) to the back-calculated scattering pattern of the symmetrical Neu5Ac-free (apo) dimer (shown in cartoon, blue), suggesting that the Neu5Ac-free structure is a reasonable model for NanR in solution. The scattering data for NanR in the presence of Neu5Ac (beige) is best fit (red line, X^2=6.6) to the back-calculated scattering pattern for the asymmetric crystal structure solved in this study (shown in cartoon, blue and beige), supporting a conformational change upon Neu5Ac binding. In contrast, the back-calculated scattering pattern of the symmetrical Neu5Ac-bound model gave a X^2=11.3 (blue line). **b,** Small angle X-ray scattering data for the NanR-dimer$_1$/DNA hetero-complex (green). The back-calculated scattering pattern based on the cryo-EM NanR-dimer$_1$/DNA hetero-complex structure (shown in cartoon, green) fits well to the experimental scattering data (grey, X^2 =2.3), suggesting that the cryo-EM model is analogous to the solution structure. **c,** The pairwise distribution plots, calculated from the scattering data, estimate the maximum inter-particle dimension (D_{max}) for NanR (105 Å, blue), NanR in the presence of Neu5Ac (101 Å, beige) and the NanR-dimer$_1$/DNA hetero-complex (98 Å, green). For all data, the Guinier plots, inset (respective colors), are linear, showing that the sample is free from aggregation or inter-particle interference.

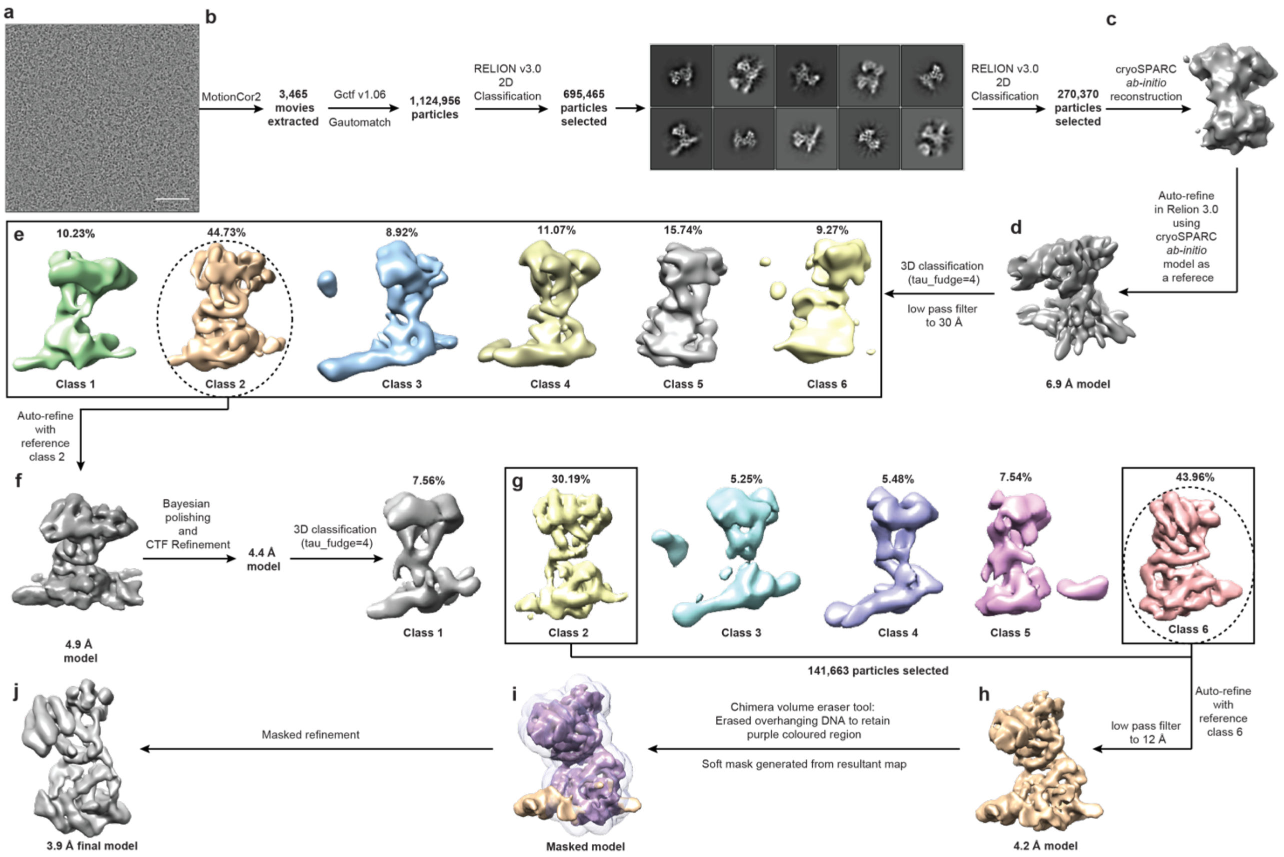

Supplementary Figure 7. Cryo-EM processing pipeline for the NanR-dimer$_1$/DNA hetero-complex. a, Electron micrograph at 2.4 µm defocus. Scale bar (white) represents 50 nm. Data is representative of 3465 independent micrographs. **b,** Two rounds of 2D classification retaining 270,370 particles that had high signal-to-noise. **c,** Initial 3D *ab initio* reconstruction. **d,** The first round of auto-refinement resulted in a 6.9 Å reconstruction. **e,** Initial 3D classification with a low pass filter to 30 Å generated a total of six discrete classes, where four of these showed features consistent with DNA binding (black box). Class 2 has the highest signal-to-noise (dash circle). The abundance of each class is shown by percentage. **f,** The second round of auto-refinement, using class 2 as a reference resulted in a 4.9 Å reconstruction. **g,** Following Bayesian polishing and CTF refinement, a second 3D classification generated a total of six discrete classes. Classes 2 and 6 have the highest signal-to-noise (black box). The abundance of each class is shown by percentage. **h,** The third round of auto-refinement, using class 6 as a reference (dash circle in **g**) and a low pass filter to 12 Å resulted in a 4.2 Å reconstruction. **i,** A soft mask was generated around the core of the model to exclude the regions with the highest flexibility (grey density). **j,** The masked refinement resulted in a final 3.9 Å reconstruction of the NanR-dimer$_1$/DNA hetero-complex.

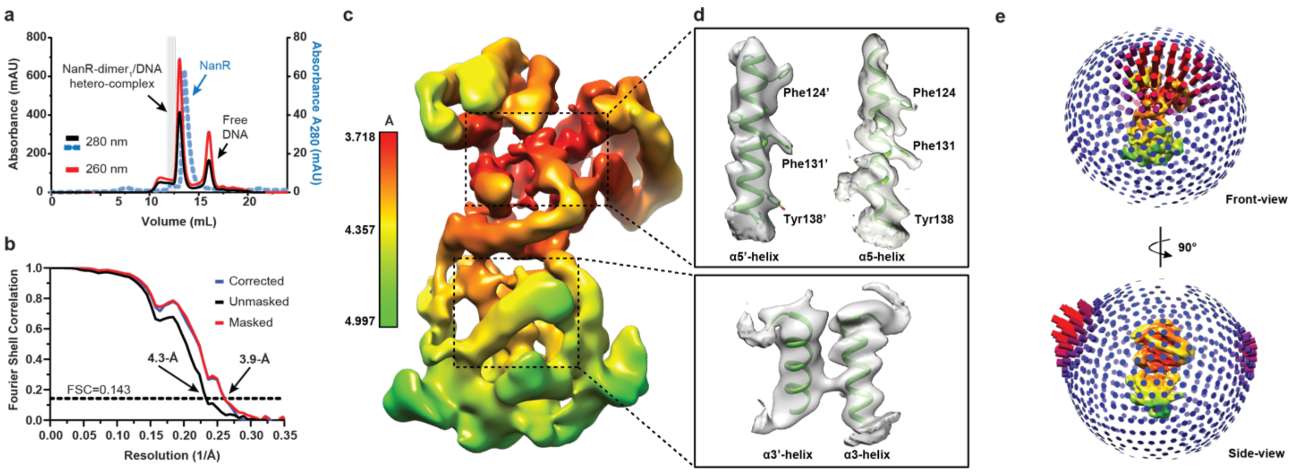

Supplementary Figure 8. Sample preparation, map quality, and preferred orientation of the NanR-dimer$_1$ cryo-EM structure. a, Size exclusion chromatogram highlighting the purification of the NanR-dimer$_1$/DNA hetero-complex from free NanR and DNA. The shaded area was pooled and subsequently used for cryo-EM sample preparation. **b,** Fourier shell correlation plot indicating a resolution of 3.9 Å and 4.3 Å for the masked (red) and unmasked (black) 3D reconstructions of the NanR-dimer$_1$/DNA hetero-complex, respectively, as reported by the FSC=0.143 criterion. **c,** Local resolution estimation for the final masked 3D reconstruction of the NanR-dimer$_1$/DNA hetero-complex. The color bar outlines the resolution range of the map in Å. **d,** α5-helices, which contribute to the dimer interface (top inset), and α3-helices, which bind DNA at the major groove (lower inset), present the highest level of resolvability. Sidechains for residues that could be assigned are labeled and shown as sticks. **e,** Angular distribution estimation for the NanR-dimer$_1$/DNA hetero-complex dataset suggests a preferential orientation of the particles. Each cylinder shown in the distribution represents one view, where the height is reflective of the number of particles in that view.

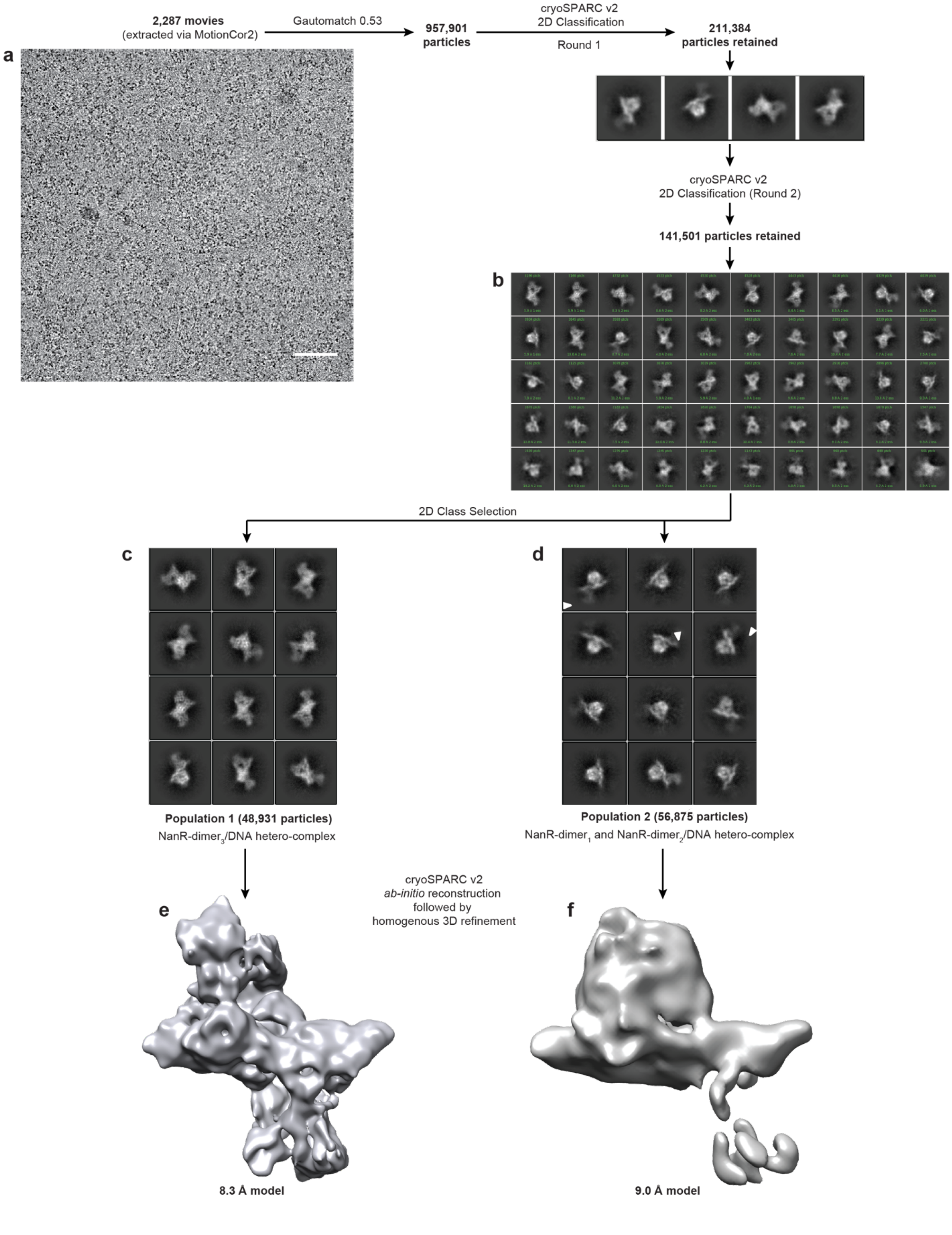

Supplementary Figure 9. Cryo-EM processing pipeline for the NanR-dimer$_3$/DNA hetero-complex. a, Electron micrograph at 2.4 µm defocus. Scale bar (white) represents 50 nm. Data is representative of 2287 independent micrographs. **b,** Two rounds of 2D classification retaining 141,501 particles that had high signal-to-noise. **c,** 2D class averages from population 1 comprised only NanR-dimer$_3$/DNA hetero-complex particle projections. **d,** 2D class averages from population 2 are consistent with a mixture of NanR-dimer$_1$ and NanR-dimer$_2$/DNA hetero-complex particle projections. The second NanR dimer is highlighted by a white arrow. **e,** Ab initio reconstruction and subsequent homogenous 3D refinement of the NanR-dimer$_3$/DNA hetero-complex dataset resulted in a final 8.3 Å reconstruction. **f,** *Ab initio* reconstruction and subsequent homogenous 3D refinement of the mixed NanR-dimer$_1$ and NanR-dimer$_2$/DNA hetero-complex dataset resulted in a final 9 Å reconstruction.

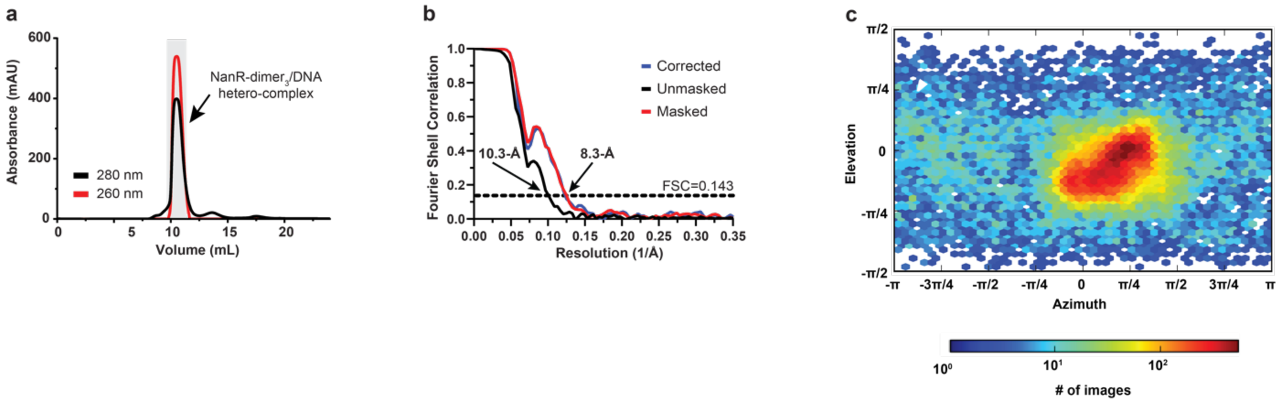

Supplementary Figure 10. Sample preparation, map quality, and preferred orientation of the NanR-dimer$_3$/DNA hetero-complex cryo-EM structure. a, Size exclusion chromatogram highlighting the purification of the NanR-dimer$_3$/DNA hetero-complex from free NanR and DNA. The shaded area was pooled and subsequently used for cryo-EM sample preparation. **b,** Fourier shell correlation plot indicating a resolution of 8.3 Å and 10.3 Å for the masked (red) and unmasked (black) 3D reconstructions of the NanR-dimer$_3$/DNA hetero-complex, respectively, as reported by the FSC=0.143 criterion. **c,** Angular distribution of NanR-dimer$_3$/DNA hetero-complex particle projections suggests a preferential orientation according to cryoSPARC v2 non-uniform refinement[14].

SUPPLEMENTARY TABLES

Supplementary Table 1 | Summary of sedimentation velocity analysis of NanR, NanR in the presence of Neu5Ac and $(GGTATA)_2$-repeat DNA.

	Wavelength (nm)	Sedimentation coefficient (S)	Molar mass (kDa)	Calculated molar mass (kDa)	Frictional ratio (f/f_0)	Fit RMSD
$(GGTATA)_3$-repeat DNA (80 nM)	495 (fluorescence)	3.00	23.0	22.6	1.68	11.3
NanR (30 µM)	280	3.70	54.5	59.0	1.32	0.005
NanR (10 µM)	280	3.65	54.2	59.0	1.32	0.004
NanR (3.3 µM)	280	3.64	54.3	59.0	1.32	0.004
NanR (10 µM) + FAM (3 µM)	495 (absorbance)	-	-	-	-	0.004
NanR (30 µM) + Neu5Ac (20 mM)	280	3.71	54.3	59.0	1.29	0.005
NanR (10 µM) + Neu5Ac (20 mM)	280	3.67	54.3	59.0	1.31	0.004
NanR (3.3 µM) + Neu5Ac (20 mM)	280	3.65	54.4	59.0	1.30	0.004
**Fixed parameters**						
Buffer density (g/cm^3)				1.006		
Buffer viscosity (cP)				1.027		
Partial specific volume (NanR)				0.7295		
Partial specific volume ($(GGTATA)_3$-repeat DNA)				0.5500		

Supplementary Table 2 | Summary of fluorescence-detection sedimentation velocity analysis of NanR titrated against $(GGTATA)_3$-repeat DNA.

Sample	Weight-averaged sedimentation coefficient (S) [Peak integration range, S]	Frictional ratio (f/f_0)	Fit RMSD
$(GGTATA)_3$-repeat DNA (80 nM)	3.00 [2.0–3.5]	1.68	11.3
0.78 nM NanR (+ 80 nM $(GGTATA)_3$-repeat DNA)	3.03 [3.5–12.0]	1.70	9.4
1.55 nM NanR (+ 80 nM $(GGTATA)_3$-repeat DNA)	3.04 [3.5–12.0]	1.71	10.0
3.1 nM NanR (+ 80 nM $(GGTATA)_3$-repeat DNA)	3.04 [3.5–12.0]	1.72	8.9
6.2 nM NanR (+ 80 nM $(GGTATA)_3$-repeat DNA)	3.07 [3.5–12.0]	1.72	10.1
12.4 nM NanR (+ 80 nM $(GGTATA)_3$-repeat DNA)	3.14 [3.5–12.0]	1.73	8.6
24.8 nM NanR (+ 80 nM $(GGTATA)_3$-repeat DNA)	3.47 [3.5–12.0]	1.86	10.0
49.6 nM NanR (+ 80 nM $(GGTATA)_3$-repeat DNA)	4.09 [3.5–12.0]	1.90	8.7
99.3 nM NanR (+ 80 nM $(GGTATA)_3$-repeat DNA)	6.00 [3.5–12.0]	1.97	10.8
198.5 nM NanR (+ 80 nM $(GGTATA)_3$-repeat DNA)	8.28 [3.5–12.0]	2.39	10.8
397.0 nM NanR (+ 80 nM $(GGTATA)_3$-repeat DNA)	8.50 [3.5–12.0]	2.43	17.0
794.0 nM NanR (+ 80 nM $(GGTATA)_3$-repeat DNA)	8.51 [3.5–12.0]	2.42	14.6
**Fixed parameters**			
Buffer density (g/cm^3)	1.006		
Buffer viscosity (cP)	1.027		
Partial specific volume (NanR)	0.7295		
Partial specific volume ($(GGTATA)_3$-repeat DNA)	0.5500		

Supplementary Table 3 | Summary of sedimentation velocity analysis of NanR and NanR$^{33\text{-}263}$ in the presence of (GGTATA)$_3$-repeat DNA.

Sample	Wavelength (nm)	Weight-averaged sedimentation coefficient (x10^{-13} S)	Frictional ratio (f/f_0)	Fit RMSD
(GGTATA)$_3$-repeat DNA (3 µM)	495	2.95	1.68	0.003
3 µM NanR (+ 3µM (GGTATA)$_3$-repeat DNA)	495	3.80	1.51	0.003
12 µM NanR (+ 3µM (GGTATA)$_3$-repeat DNA)	495	6.32	1.56	0.003
24 µM NanR (+ 3µM (GGTATA)$_3$-repeat DNA)	495	9.11	1.62	0.003
10 µM NanR$^{33\text{-}263}$	495	3.38	1.25	0.004
3 µM NanR$^{33\text{-}263}$ (+ 3µM (GGTATA)$_3$-repeat DNA)	495	3.43	1.61	0.003
12 µM NanR$^{33\text{-}263}$ (+ 3µM (GGTATA)$_3$-repeat DNA)	495	4.39	1.53	0.003
24 µM NanR$^{33\text{-}263}$ (+ 3µM (GGTATA)$_3$-repeat DNA)	495	4.45	1.49	0.003
**Fixed parameters**				
Buffer density (g/cm^3)		1.006		
Buffer viscosity (cP)		1.027		
Partial specific volume (NanR)		0.7295		
Partial specific volume (NanR$^{33–263}$)		0.7300		
Partial specific volume ((GGTATA)$_3$-repeat DNA)		0.5500		

Supplementary Table 4 | Integration results from multi-wavelength sedimentation velocity analysis of deconvoluted NanR$^{33\text{-}263}$ and (GGTATA)$_3$-repeat DNA sedimentation profiles.

	NanR$^{33\text{-}263}$ only	(GGTATA)$_3$-repeat DNA only	NanR$^{33\text{-}263}$:DNA loading ratio 3:1	10:1
Sed. Coefficient ($\times 10^{-13}$ S) *	3.42 (3.09, 3.77)	2.76 (2.63, 2.89)	4.39 (4.10, 4.67)	4.46 (3.58, 5.33)
Dif. Coefficient ($\times 10^{-7}$ D) *	6.79 (4.09, 9.49)	6.99 (6.10, 7.86)	4.97 (4.03, 5.91)	5.04 (3.39, 6.70)
Measured molar ratio ‡	n/a	n/a	2.44	2.24
Weight-averaged $\bar{v}$ (mL g^{-1}) §	0.730	0.55	0.677	0.677
Measured molar mass (kDa) ¶	45.3 (29.7, 62.8)	21.4 (17.9, 24.8)	66.3	66.4
Theoretical molar mass (kDa) #	51.3	21.5	72.8	72.8
Oligomeric state of hetero-complex	n/a	n/a	NanR-dimer$_1$/DNA	NanR-dimer$_1$/DNA

* Sedimentation and diffusion coefficients observed following 2DSA-Monte Carlo analysis. Parameters are obtained from integration of pseudo-3D plots using UltraScan[15]. All measured values represent the mean from the Monte Carlo analysis. The values in parentheses are the 95% confidence intervals from the Monte Carlo analysis.

‡ Partial concentration is determined from peak integration of the co-migrating species in both the NanR and DNA datasets. Because the data is scaled to molar concentrations, the molar ratio and thus stoichiometry of the hetero-complex can be inferred.

§ Partial specific volume ($\bar{v}$) of the hetero-complex, estimated from the weight-average of the protein and DNA components. A $\bar{v}$ of 0.7295 mL g^{-1} was used for NanR, while a $\bar{v}$ of 0.55 mL g^{-1} was used for DNA. The equation used to calculate the weight-averaged $\bar{v}$ is presented in the Methods.

¶ The measured molar mass is estimated based on the hydrodynamic parameters (sedimentation and diffusion coefficient) and the weighted-averaged $\bar{v}$, calculated using the measured molar ratio. The values in parentheses are the 95% confidence intervals from the Monte Carlo analysis for the pure components.

\# The theoretical mass is predicted based upon the amino acid or nucleic acid sequence within UltraScan[15]. The mass of each hetero-complex is predicted based on the observed molar ratio.

Supplementary Table 5 | Integration results from multi-wavelength sedimentation velocity analysis of deconvoluted NanR and $(GGTATA)_3$-repeat DNA sedimentation profiles.

	**NanR only**	**$(GGTATA)_3$-repeat DNA only**	**NanR:DNA loading ratio**						
			**1:1** (Species 1)	**1:1** (Species 2)	**3:1** (Species 1)	**3:1** (Species 2)	**6:1** (Species 2)	**6:1** (Species 3)	**10:1** (Species 3)
Sed. Coefficient ($\times 10^{-13}$ S) *	3.72 (3.50, 3.94)	2.76 (2.63, 2.89)	5.67 (5.09 6.26)	7.45 (7.01, 7.90)	5.62 (4.83, 6.41)	7.46 (6.82, 8.11)	7.54 (7.13, 7.95)	7.99 (7.35, 8.61)	8.31 (7.95, 8.68)
Dif. Coefficient ($\times 10^{-7}$ D) *	6.3 (3.84, 8.76)	6.99 (6.10, 7.86)	4.37 (2.46, 6.30)	N/D†	4.40 (2.18, 6.11)	N/D †	N/D †	3.16 (2.65, 3.66)	3.40 (2.49, 4.31)
Measured molar ratio ‡	n/a	n/a	2.45	4.25	2.37	3.63	4.65	6.44	6.21
Weight-averaged $\bar{\upsilon}$ (mL g^{-1}) §	0.730	0.550	0.682	0.702	0.682	0.702	0.702	0.710	0.710
Measured molar mass (kDa) ¶	54.0 (30.0, 77.9)	21.4 (17.9, 24.8)	98.9	N/D†	97.6	N/D†	N/D†	211.8	204.8
Theoretical molar mass (kDa) #	59.0	21.5	80.5	139.5	80.5	139.5	139.5	198.5	198.5
Oligomeric state of hetero-complex	n/a	n/a	NanR-dimer$_1$/ DNA	NanR-dimer$_2$/ DNA	NanR-dimer$_1$/ DNA	NanR-dimer$_2$/ DNA	NanR-dimer$_2$/ DNA	NanR-dimer$_3$/ DNA	NanR-dimer$_3$/ DNA

* Sedimentation and diffusion coefficients observed following 2DSA-Monte Carlo analysis. Parameters are obtained from integration of pseudo-3D plots using UltraScan[15]. All measured values represent the mean from the Monte Carlo analysis. The values in parentheses are the 95% confidence intervals from the Monte Carlo analysis.

† N/D = not determined as species were present within a reaction boundary.

‡ Partial concentration is determined from peak integration of the co-migrating species in both the NanR and DNA datasets. Because the data is scaled to molar concentrations, the molar ratio and thus stoichiometry of the hetero-complex can be inferred.

§ Partial specific volume ($\bar{\upsilon}$) of the hetero-complex, estimated from the weight-average of the protein and DNA components. A $\bar{\upsilon}$ of 0.7295 mL g^{-1} was used for NanR, while a $\bar{\upsilon}$ of 0.55 mL g^{-1} was used for DNA. The equation used to calculate the weight-averaged $\bar{\upsilon}$ is presented in the Methods.

¶ The measured molar mass is estimated based on the hydrodynamic parameters (sedimentation and diffusion coefficient) and the weighted-averaged $\bar{\upsilon}$, calculated using the measured molar ratio. The values in parentheses are the 95% confidence intervals from the Monte Carlo analysis (pure controls).

\# The theoretical mass is predicted based upon the amino acid or nucleic acid sequence within UltraScan[15]. The mass of each hetero-complex is predicted based on the observed molar ratio.

Supplementary Table 6 | Summary of fluorescence-detection sedimentation velocity analysis of NanR titrated against DNA in the presence of Neu5Ac.

Sample	Weight-averaged sedimentation coefficient (S) [Peak integration range, S]	Frictional ratio (f/f_0)	Fit RMSD
(GGTATA)$_3$-repeat DNA (80 nM)	2.93 [2.0–3.5]	1.68	4.85
0.78 nM NanR (+ 80 nM (GGTATA)$_3$-repeat DNA)	2.94 [3.5–12.0]	1.67	5.27
1.55 nM NanR (+ 80 nM (GGTATA)$_3$-repeat DNA)	2.93 [3.5–12.0]	1.69	4.85
3.1 nM NanR (+ 80 nM (GGTATA)$_3$-repeat DNA)	2.93 [3.5–12.0]	1.69	5.12
6.2 nM NanR (+ 80 nM (GGTATA)$_3$-repeat DNA)	2.94 [3.5–12.0]	1.68	4.89
12.4 nM NanR (+ 80 nM (GGTATA)$_3$-repeat DNA)	2.92 [3.5–12.0]	1.68	5.15
24.8 nM NanR (+ 80 nM (GGTATA)$_3$-repeat DNA)	2.97 [3.5–12.0]	1.69	4.92
49.6 nM NanR (+ 80 nM (GGTATA)$_3$-repeat DNA)	2.97 [3.5–12.0]	1.69	4.75
99.3 nM NanR (+ 80 nM (GGTATA)$_3$-repeat DNA)	3.02 [3.5–12.0]	1.69	5.01
198.5 nM NanR (+ 80 nM (GGTATA)$_3$-repeat DNA)	3.46 [3.5–12.0]	1.74	4.75
397.0 nM NanR (+ 80 nM (GGTATA)$_3$-repeat DNA)	4.78 [3.5–12.0]	1.88	4.83
794.0 nM NanR (+ 80 nM (GGTATA)$_3$-repeat DNA)	7.84 [3.5–12.0]	1.99	5.23
**Fixed parameters**			
Buffer density (g/cm^3)			
Buffer viscosity (cP)			
Buffer density (with 20 mM Neu5Ac) (g/cm^3)			
Buffer viscosity (with 20 mM Neu5Ac) (cP)			
Partial specific volume (NanR)			
Partial specific volume ((GGTATA)$_2$-repeat DNA)			

Supplementary Table 7 | X-ray crystallography data collection, refinement and validation statistics*

**Data collection parameters**	**C-terminal substructure**	**NanR** (PDB-60N4)
Beamline	MX2	MX2
Detector	EIGER x 16M	EIGER x 16M
X-ray wavelength (Å)	1.2781	0.9537
Crystal-to-detector distance (mm)	245	200
Space group	$I\,2_1\,2_1\,2_1$	$P\,2_1$
Unit cell *a*, *b*, *c* (Å)	75.46, 78.35, 88.72	39.77, 87.85, 73.87
Unit cell α, β, γ (°)	90, 90, 90	90, 103, 90
Resolution range (Å)	44.36–2.29 (2.35–2.29)	43.9–2.10 (2.17–2.10)
No. of total reflections	1,084,309	96,673
No. of unique reflections	22,937	28,482
Completeness (%)	99.6 (97.9)	98.1 (88.3)
R_{merge} (%)	7.7 (49.2)	4.4 (53.5)
CC_{anom}	44(1)	0(0)
$CC_{1/2}$ (%)	99.9 (95.2)	99.9 (69.4)
$I/\sigma(I)$	35.9(5.10)	15.3 (1.6)
**Refinement statistics**		
Resolution (Å)		43.9–2.10
No. of reflections used		26,874
No. of reflection used in test set		1,368
R_{work}/R_{free} (%)		18.12/22.95
Total non-H atoms		3,653
Protein/Water		3,586
Zn^{2+}		1
Neu5Ac		66
Mean *B* factor ($Å^2$)		
Protein		56.5
Zn^{2+}		38.8
Neu5Ac		37.3
Waters/PEG4K		68.1
r.m.s deviations		
Bond (Å)		0.009
Angle (°)		1.27
***MolProbity* statistics†**		
Rotamer outliers (%)		3.88
Clashscore		9.17
Ramachandran plot		
Favored/allowed regions (%)		98.36/1.64

* Values in parentheses are for the highest resolution shell

† *MolProbity* structure-validation web service[16]

Supplementary Table 8 | Data collection and analysis statistics from small-angle X-ray scattering experiments.

**Analysis statistics**	**NanR**	**NanR (+ 20 mM Neu5Ac)**	**NanR-dimer$_1$/DNA hetero-complex**
$I(0)$ (cm^{-1}) (from Guinier analysis)	$0.033 \pm 8.9e^{-05}$	$0.032 \pm 8.9e^{-05}$	$0.1 \pm 5.5e^{-04}$
R_g (Å) (from Guinier analysis)	32.3 ± 0.3	31.4 ± 0.2	33.4 ± 0.6
$I(0)$ (cm^{-1}) (from $P(r)$ analysis)	$0.033 \pm 0.1e^{-03}$	$0.032 \pm 0.1e^{-03}$	$0.010 \pm 0.9e^{-03}$
R_g (Å) (from $P(r)$ analysis)	32.5 ± 0.2	31.6 ± 0.2	32.8 ± 0.2
D_{max} (Å)	105	101	98
Porod volume ($Å^{-3}$)	110,038	104,710	108,102
Molar mass (from Porod Volume, kDa)	64.7	61.6	63.6
Molar mass (from SAXSMoW2*, kDa)	70.6	63.6	82.9
Calculated dimer MM from sequence (kDa)	59.0	59.0	70.5
**Data collection parameters**			
Instrument	Australian Synchrotron SAXS/WAXS beamline		
Detector	PILATUS 1M (Dectris)		
Wavelength (Å)	1.0332		
Maximum flux at sample	8×10^{12} photons per second at 12 keV		
Camera length (mm)	1600		
q range ($Å^{-1}$)	0.006–0.5		
Exposure time	Continuous 1 second frame measurements		
Sample configuration	SEC-SAXS with co-flow		
Sample temperature (°C)	12		

[a] http://saxs.ifsc.usp.br/[17]

Supplementary Table 9 | Cryo-EM data collection, refinement and validation statistics.

**Data collection parameters**	**NanR-dimer$_1$/DNA hetero-complex** (EMD-21652, PDB-6WFQ)	**NanR-dimer$_3$/DNA hetero-complex** (EMD-21661, PDB-6WG7)
Molecular mass (kDa)	70.5	198.5
Magnification	215,000 (EFTEM)	150,000
Voltage (kV)	300	200
Camera	Gatan K2	FEI Falcon3
Dose rate (e/pixel/s)	4	0.8
Electron exposure ($e^{-}Å^{-2}$)	60	45
Defocus range (µm)	-0.5 to -2.5	-0.5 to -1.5
Pixel size (Å)	0.68	0.94
Symmetry imposed	C1	C1
Initial particle images (no.)	695,465	957,901
Final particle images (no.)	141,663	48,931
Relative abundance (%)[a]	20.39	5.11
Applied map sharpening B factor ($Å^2$)	-100	-250
Map resolution (Å)	3.9	8.3
FSC threshold	0.143	0.143
Map resolution range (Å)	3.710 to 6.096	N/A
**Refinement statistics**		
Model composition		
Non-hydrogen atoms	4164	
Protein residues	447	
Nucleotide residues	30	
r.m.s deviations		
Bond lengths (Å)	0.011	
Bond angles (°)	1.159	
**Validation statistics**		
MolProbity† score	2.9	
Rotamer outliers (%)	1.9	
Clashscore, all atoms	49.6	
Ramachandran plot		
Favored (%)	96.0	
Allowed (%)	4.0	
Disallowed (%)	0.0	

[a] Relative abundance with respect to the total number of images in the initial cryo-EM dataset.

† *MolProbity* is a structure-validation web service[16]

Supplementary Table 10 | Oligonucleotide and NanR template sequences

NanR NdeI 33 fwd†	5’-aaggagatataCATATGctctccgaaatggtggaagaag-3’
NanR 263 stop HindIII rev	5’-gtgcggccgcAAGCTTttatttctttttgttggtggtctg-3’
NanR sequence	MGLMNAFDSQTEDSSPAIGRNLRSRPLARKKLSEMVEEELEQMIRRREFGEGEQLPSERE LMAFFNVGRPSVREALAALKRKGLVQINNGERARVSRPSADTIIGELSGMAKDFLSHPGG IAHFEQLRLFFESSLVRYAAEHATDEQIDLLAKALEINSQSLDNNAAFIRSDVDFHRVLA EIPGNPIFMAIHVALLDWLIAARPTVTDQALHEHNNVSYQQHIAIVDAIRRHDPDEADRA LQSHLNSVSATWHAFGQTTNKKK

† Restriction sites underlined

SUPPLEMENTARY DATA REFERENCES

1. Schuck, P. Size-distribution analysis of macromolecules by sedimentation velocity ultracentrifugation and lamm equation modeling. *Biophys. J.* **78**, 1606-19 (2000).
2. Sievers, F. et al. Fast, scalable generation of high-quality protein multiple sequence alignments using Clustal Omega. *Mol. Syst. Biol.* **7**(2011).
3. Robert, X. & Gouet, P. Deciphering key features in protein structures with the new ENDscript server. *Nucleic Acids Res.* **42**, W320-W324 (2014).
4. Little, M.S., Pellock, S.J., Walton, W.G., Tripathy, A. & Redinbo, M.R. Structural basis for the regulation of beta-glucuronidase expression by human gut Enterobacteriaceae. *Proc Natl Acad Sci U S A* (2017).
5. van Aalten, D.M., DiRusso, C.C., Knudsen, J. & Wierenga, R.K. Crystal structure of FadR, a fatty acid-responsive transcription factor with a novel acyl coenzyme A-binding fold. *EMBO J.* **19**, 5167-77 (2000).
6. Gao, Y.G. et al. Structural and functional characterization of the LldR from *Corynebacterium glutamicum*: a transcriptional repressor involved in L-lactate and sugar utilization. *Nucleic Acids Res.* **36**, 7110-23 (2008).
7. Lord, D.M. et al. McbR/YncC: implications for the mechanism of ligand and DNA binding by a bacterial GntR transcriptional regulator involved in biofilm formation. *Biochemistry* **53**, 7223-31 (2014).
8. Zheng, M. et al. Structure of *Thermotoga maritima* TM0439: implications for the mechanism of bacterial GntR transcription regulators with Zn2+-binding FCD domains. *Acta Crystallogr., Sect. D: Biol. Crystallogr.* **65**, 356-65 (2009).
9. Gorbet, G.E., Pearson, J.Z., Demeler, A.K., Colfen, H. & Demeler, B. Next-Generation AUC: Analysis of Multiwavelength Analytical Ultracentrifugation Data. in *Analytical Ultracentrifugation*, Vol. 562 (ed. Cole, J.L.) 27-47 (2015).
10. Lawson, C.L. & Hanson, R.J. *Solving Least Squares Problems*, (Prentice-Hall, Inc., Englewood Cliffs, NJ., 1974).
11. Handing, K.B. et al. Characterizing metal-binding sites in proteins with X-ray crystallography. *Nat. Protoc.* **13**, 1062-1090 (2018).
12. Evans, G. & Pettifer, R.F. CHOOCH: a program for deriving anomalous-scattering factors from X-ray fluorescence spectra. *J. Appl. Crystallogr.* **34**, 82-86 (2001).
13. Laskowski, R.A. & Swindells, M.B. LigPlot+: multiple ligand-protein interaction diagrams for drug discovery. *J. Chem. Inf. Model.* **51**, 2778-86 (2011).
14. Punjani, A., Rubinstein, J.L., Fleet, D.J. & Brubaker, M.A. cryoSPARC: algorithms for rapid unsupervised cryo-EM structure determination. *Nat. Methods* **14**, 290-296 (2017).
15. Demeler, B. UltraScan - A Comprehensive Data Analysis Software Package for Analytical Ultracentrifugation Experiments. in *Analytical Ultracentrifugation: Techniques and Methods* (eds. Scott, D.J., Harding, S.E. & Rowe, A.J.) 210-230 (Royal Society of Chemistry, UK, 2005).
16. Chen, V.B. et al. MolProbity: all-atom structure validation for macromolecular crystallography. *Acta Crystallogr. D* **66**, 12-21 (2010).
17. Fischer, H., Neto, M.D., Napolitano, H.B., Polikarpov, I. & Craievich, A.F. Determination of the molecular weight of proteins in solution from a single small-angle X-ray scattering measurement on a relative scale. *J. Appl. Crystallogr.* **43**, 101-109 (2010).
