# Supplementary material for: Mechanism of NanR gene repression and allosteric induction of bacterial sialic acid metabolism
Source: Nat Commun. 2021 Mar 31;12:1988. doi: 10.1038/s41467-021-22253-6 (PMC8012715; doi:10.1038/s41467-021-22253-6)
Supplement: Supplementary file 3 — Description of Additional Supplementary Files [file 41467_2021_22253_MOESM3_ESM.pdf]

Description of Additional Supplementary Files

File Name: Supplementary Movie 1
Description: X-ray crystal structure of NanR in complex with Neu5Ac and Zn^{2+} and closeup of the effector binding site.
Accompaniment to Fig. 4b-c.

File Name: Supplementary Movie 2
Description: Cryo-EM structure of the NanR-dimer1/DNA hetero-complex and closeup on the protein-DNA interface.
Accompaniment to Fig. 5.

File Name: Supplementary Movie 3
Description: Morph model to illustrate the conformational change between the DNA-free (X-ray crystal structure) and the DNA-bound (cryo-EM structure) states.
Accompaniment to Fig. 6.

File Name: Supplementary Movie 4
Description: Cryo-EM structure of the NanR-dimer3/DNA hetero-complex and closeup of each dimer assembled across the (GGTATA)3-repeat operator DNA.
Accompaniment to Fig. 7.
