# Supplementary material for: Mechanism of NanR gene repression and allosteric induction of bacterial sialic acid metabolism
Source: Nat Commun. 2021 Mar 31;12:1988. doi: 10.1038/s41467-021-22253-6 (PMC8012715; doi:10.1038/s41467-021-22253-6)
Supplement: Supplementary file 8 — Reporting Summary [file 41467_2021_22253_MOESM8_ESM.pdf]

nature research

Corresponding author(s): Prof. Renwick Dobson

Last updated by author(s): Feb 28, 2021

Reporting Summary

Nature Research wishes to improve the reproducibility of the work that we publish. This form provides structure for consistency and transparency in reporting. For further information on Nature Research policies, see our Editorial Policies and the Editorial Policy Checklist.

Statistics

For all statistical analyses, confirm that the following items are present in the figure legend, table legend, main text, or Methods section.

n/a	Confirmed	
☐	☒	The exact sample size (*n*) for each experimental group/condition, given as a discrete number and unit of measurement
☐	☒	A statement on whether measurements were taken from distinct samples or whether the same sample was measured repeatedly
☒	☐	The statistical test(s) used AND whether they are one- or two-sided<br>*Only common tests should be described solely by name; describe more complex techniques in the Methods section.*
☒	☐	A description of all covariates tested
☒	☐	A description of any assumptions or corrections, such as tests of normality and adjustment for multiple comparisons
☐	☒	A full description of the statistical parameters including central tendency (e.g. means) or other basic estimates (e.g. regression coefficient) AND variation (e.g. standard deviation) or associated estimates of uncertainty (e.g. confidence intervals)
☒	☐	For null hypothesis testing, the test statistic (e.g. F, t, r) with confidence intervals, effect sizes, degrees of freedom and P value noted<br>*Give P values as exact values whenever suitable.*
☒	☐	For Bayesian analysis, information on the choice of priors and Markov chain Monte Carlo settings
☒	☐	For hierarchical and complex designs, identification of the appropriate level for tests and full reporting of outcomes
☒	☐	Estimates of effect sizes (e.g. Cohen's d, Pearson's r), indicating how they were calculated

Our web collection on statistics for biologists contains articles on many of the points above.

Software and code

Policy information about availability of computer code

Data collection	ProteomeLab XL-I (ver. 1) and OptimaAUC (ver. 1) was used to acquire analytical ultracentrifugation data.<br>Nano ITCRun (ver. 3.7.0) was used to acquire isothermal calorimetry data.<br>EPU software package (Thermo Fisher Scientific, ver. 2.6) was used for all automated cyro-electron microscopy data collection and autofocus.<br>ScattterBrain (Linux, ver. 2.8.2; Australian Synchrotron) was used for to radially average, normalize against sample transmission and background-substract SAXS data.
Data analysis	SEDFIT (ver. 16.2c), SEDNTERP (ver. 3) and UltraScan (ver. 4.0, release 2843) was used to analyze analytical ultracentrifugation data.<br>ImageJ (ver. 1.8) was used to analyze the EMSA experiments.<br>GraphPad Prism (ver. 8.3.0) was used for the nonlinear regression (curve fit).<br>NITPIC (version 1.2.7) and SEDPHAT (version 15-2b) was used to analyze isothermal calorimetry data.<br>XDS package (includes XDS_NONISOMORPHISM and XSCALE-ver. march 15, 2019), CCP4 suite (ver. 7.1), SHELXC/D/E package (ver. 1.0.1225), ABS program (intergrated into CCP4 suite), RESOLVE (ver. 2.13), ARP/wARP (ver. 8.0), MOLPROBITY (ver. 4.5.1), Phenix software package (ver. 1.17.1-3660), RELION (ver. 3.0), cryoSPARC (ver. 2), and COOT (ver. 0.8.9.2) were used for X-ray crystallography and Cryo-EM structure determination.<br>PDBePISA (ver. 1.48) was used to analyze the protein oligomeric interface.<br>The ATSAS software package (ver. 3.0) was used to analyze SAXS data.<br>PyMOL (ver. 2.3.3) and UCSF Chimera (ver. 1.14) was used to visualise and interpret all structural data.

For manuscripts utilizing custom algorithms or software that are central to the research but not yet described in published literature, software must be made available to editors and reviewers. We strongly encourage code deposition in a community repository (e.g. GitHub). See the Nature Research guidelines for submitting code & software for further information.

Data

Policy information about availability of data

All manuscripts must include a data availability statement. This statement should provide the following information, where applicable:

- Accession codes, unique identifiers, or web links for publicly available datasets
- A list of figures that have associated raw data
- A description of any restrictions on data availability

Data are available from the corresponding author upon reasonable request. The atomic models for NanR bound to Neu5Ac, the NanR-dimer1/DNA hetero-complex and the NanR-dimer3/DNA hetero-complex are available through the Protein Data Bank with the accession codes 6ON4 [http://doi.org/10.2210/pdb6ON4/pdb], 6WFQ [http://doi.org/10.2210/pdb6WFQ/pdb] and 6WG7 [http://doi.org/10.2210/pdb6WG7/pdb], respectively. Cryo-EM reconstructions of the NanR-dimer1/DNA hetero-complex and NanR-dimer3/DNA hetero-complex are available through the Electron Microscopy Data Bank with accession codes EMDB-21652 [https://www.ebi.ac.uk/pdbe/entry/emdb/EMD-21652] and EMDB-21661 [https://www.ebi.ac.uk/pdbe/entry/emdb/EMD-21661], respectively. Small angle X-ray scattering data for NanR, NanR in the presence of Neu5Ac and the NanR-dimer1/DNA hetero-complex are available through the Small Angle Scattering Biological Data Bank with accession codes SASDHR9 [https://www.sasbdb.org/data/SASDHR9/], SASDHS9 [https://www.sasbdb.org/data/SASDHS9/] and SASDHT9 [https://www.sasbdb.org/data/SASDHT9/], respectively. The nanR gene is available through the UniProt database with the accession code (PA08W0) [https://www.uniprot.org/uniprot/P0A8W0]. Source data are provided with this paper

Field-specific reporting

Please select the one below that is the best fit for your research. If you are not sure, read the appropriate sections before making your selection.

[x] Life sciences   [ ] Behavioural & social sciences   [ ] Ecological, evolutionary & environmental sciences

For a reference copy of the document with all sections, see nature.com/documents/nr-reporting-summary-flat.pdf

Life sciences study design

All studies must disclose on these points even when the disclosure is negative.

Sample size	Protein expressions/preparations and EMSA gels were repeated a minimum of three times with reproducible results. Analytical ultracentrifugation runs are representative of one biological sample, where the analysis is validated by the randomly distributed residuals and low RMSD (shown in Supplementary Tables 1 to 6). The binding isotherm data are representative of one independent experiment, where concordant data were obtained from complementary techniques. ITC experiments were performed in duplicate to ensure reproducibility. Sample sizes are stated in legends.
Data exclusions	Nil. All data is presented
Replication	Number of replicates is stated in figure legends
Randomization	This is not relevant to our study. Our study compared the biophysical properties of purified wild-type and mutant NanR in parallel or under the same experimental setup
Blinding	This is not relevant to our study. Our study compared the biophysical properties of purified wild-type and mutant NanR in parallel or under the same experimental setup

Reporting for specific materials, systems and methods

We require information from authors about some types of materials, experimental systems and methods used in many studies. Here, indicate whether each material, system or method listed is relevant to your study. If you are not sure if a list item applies to your research, read the appropriate section before selecting a response.

Materials & experimental systems

n/a	Involved in the study
[x]	[ ] Antibodies
[x]	[ ] Eukaryotic cell lines
[x]	[ ] Palaeontology and archaeology
[x]	[ ] Animals and other organisms
[x]	[ ] Human research participants
[x]	[ ] Clinical data
[x]	[ ] Dual use research of concern

Methods

n/a	Involved in the study
[x]	[ ] ChIP-seq
[x]	[ ] Flow cytometry
[x]	[ ] MRI-based neuroimaging
